# Common mental disorders and its determinants among epileptic patients at an outpatient epileptic clinic in Felegehiwot Referral Hospital, Bahirdar, Ethiopia: cross-sectional study

**DOI:** 10.1186/s13033-019-0333-4

**Published:** 2019-12-28

**Authors:** Mengesha Birkie Wubie, Mogesie Necho Alebachew, Asmare Belete Yigzaw

**Affiliations:** Department of Psychiatry, College of Medicine and Health Sciences, Wollo University, Dessie, Ethiopia

**Keywords:** Common mental disorders, Factors, Epilepsy, Ethiopia

## Abstract

**Background:**

Epilepsy is a chronic neurological condition that is highly predisposed to a variety of mental health problems due to its huge biological, social and psychological burdens. Despite this, there is a paucity of research in this area. Therefore, assessing common mental disorders and its determinants among epileptic patients would be of great importance.

**Objective:**

This study was aimed to asses prevalence and associated factors of common mental disorders among people with epilepsy attending Felegehiwot Referral Hospital, Bahirdar, Ethiopia, 2019.

**Methods:**

Institutional based analytic cross-sectional study design was utilized from January to February 2019 at Felegehiwot Referral Hospital among 422 epileptic patients who were diagnosed clinically and on follow up treatment. Systematic random sampling was applied to recruit participants. Interviewer based and pretested Self Reporting Questionnaire‐20 was used to screen common mental illness with a cut-off point 7 and above as having a common mental disorder. Bivariate and multivariable logistic regression analysis with 95% CI were computed and variables with p < 0.05 in the final model were considered as associated factors for common mental disorders.

**Result:**

Four hundred twenty-two patients with epilepsy were included in our study with a response rate of 100% and 64.5% were males. The mean age of participants was 59 ± 13.37 years. Common mental disorder among epileptic patients was found to be 35.8%. There was a statistically significant association between marital status, comorbid medical illness, nicotine dependence, alcohol misuse, and medication non-adherence with common mental disorders at p < 0.05.

**Conclusion:**

The prevalence of common mental distress was high (35.8%) suggesting that it is a public health issue. Marital status, comorbid medical illness, nicotine dependence, alcohol misuse, and medication non-adherence were the factors having an association with a common mental disorder. Therefore, early screening and recognition of mental distress symptoms should be a routine activity while managing epileptic patients.

## Background

Epilepsy is defined as a neurological condition that is characterized by two or more unprovoked seizures according to the international league against epilepsy [1]. It is a chronic neurological condition estimated to affect 50 million people worldwide according to WHO [2]. Despite the worldwide prevalence of active epilepsy varies from 0.2 to 4.1% [3], higher prevalence and incidence are from low and middle-income countries (LMICs) when compared with developed countries [4, 5]. In fact, more than 85% of the global burden of epilepsy occurs in people living in LMICs [6, 7]. Epilepsy poses a multidimensional effect on the body like physical, mental and behavioral functions limitations and is associated with great risk of premature mortality due to factors like a traumatic injury to the brain, status epilepticus, suicide, pneumonia and sudden death [8, 9].

Common mental disorders define a range of anxiety and depressive disorders. Globally, 150 million individuals are affected by depression and 1 million commit suicide every year. Four of the top six causes of years lived with disability are due to neuropsychiatric disorders and more than 90 million suffer from alcohol or other substance use disorders [10, 11]. Projections to 2030 indicate that depression will be the leading cause of disability globally accounting for 6% of all [12]. The global rate of prevalence for depression ranges from 3.6 to 5.4% in the Western Pacific and African Region respectively. Whereas, anxiety disorders, rates from 2.9% in the Western Pacific Region to 5.8% in the Region of the Americas [13].

Studies done reported that a high prevalence of psychiatric disorders in people with epilepsy [14–16] and a higher risk of psychopathology compared with the general population or people with chronic non-neurological disorders [17]. Most (88%) of the patients experienced psychiatric disturbances and the most common psychiatric symptoms were neurovegetative (62%), anxiety (45%), and depression (43%), cognitive difficulties 82% [18]. Other studies showed that the prevalence of psychiatric disorders was 70% in Brazil [19], 25% for anxiety disorders, 19% for mood disorder in Dutch [20], 5.9% in Sweden [21], 52% in Iceland [22], 34.2% for mood disorder and 22.8% for anxiety [14], 50% of patients with epilepsy in United States [23].

Risk factors for common mental disorders among epileptic patients were family history of psychiatric illness [24], comorbidity of medical condition [25], being female, young age, lower-income, worse QOLIE-89 scores, more adverse drug events, unemployment [26], high seizure frequency, and low educational status in southwest Ethiopia [27], duration of illness, seizure frequency, poly-pharmacy, difficulties of adherence to antiepileptic drugs in northwest Ethiopia [28].

Common mental disorders reduce health-related quality of life (HRQOL) in patients with epilepsy [29, 30]. Comorbid depression in epileptic patients had socio-economic and physical consequences like disability in the social domain, cost of drug use and premature death [31, 32]. In addition, it affects the cognitive function of epileptic patients especially if they are not treated early or timely with the antiepileptic drug [33] and common mental disorder increases the risk of developing adverse effects of anti-epileptic medications [34]. Besides, anti-epileptic drugs have a psychotropic effect that affects the brain negatively [35]. Moreover, the suicide rate in epilepsy is five times higher and depression will be a risk factor for seizure [36].

Despite this, there is a paucity of research into this area, especially in the Ethiopian context. Even those studies have done so fare focused mainly on a specific mental disorder like depression. As per the investigators’ knowledge, there is no study done regarding the prevalence and associated factors of common mental disorders among epileptic patients in the study area.

Therefore this study was aimed to asses common mental disorders in epileptic patients and its associated factors, which would fill the gap in information by generating updated information and services as baseline evidence for future researchers and policymakers.

## Methods and materials

### Study design and setting

Institution based analytical cross-sectional study was implemented at Felegehiwot Referral Hospital, Bahirdar, Ethiopia from January to February 2019. This study was conducted at the Epileptic clinic of Felegehiwot Referral Hospital. The hospital is located in Bahirdar; which is the Capital City of the Amhara Region. The hospital services a catchment population of more than 5.5 million people [37].

### Study participants

Participants were all epileptic patients registered for follow up of anti-epileptic medication in the Epileptic clinic of Felegehiwot Referral Hospital and as evidenced from the registration book, more than 3150 patients have a history of follow up for epilepsy in the clinic. On average 225 epileptic clients visit the clinic weekly so that the total number of epileptic patients estimated to attended follow up during the data collection period is 900. The optimal size of the sample had been calculated using a single population proportion formula; by taking the prevalence of common mental disorder 49.3% from a study conducted in Jimma [38] with a 5% margin of error and 95% confidence interval of certainty (alpha = 0.05) and 10% non-response. Based on these assumptions, the sample size for the study was computed to be 422.

A systematic sampling method was applied to recruit eligible participants. The sampling interval was determined by dividing the average number of epileptic patients attending outpatient follow up monthly (900) by total sample size (422) (N/n (K = 2)). The first participant had been included by the lottery method and every 2nd case of epileptic patients attending care and treatment at Felegehiwot Referral Hospital were considered for the survey.

All follow up epileptic cases in the study period whose age was 18 years and above were allowed to take part in the study whereas those Epileptic patients unable to communicate during the interview were excluded.

### Operational definitions

*Common mental disorder* A score ≥ 7 on Self-reporting questionnaires SRQ-20 [39].

*Epilepsy* In this study, epilepsy refers to a neurological condition characterized by two or more unprovoked seizures [1].

*Ever substance use* use of a specified substance for non-medical purposes at least once in their lifetime. Alcohol use problem: For alcohol use disorders CAGE was used which had a score of 0–4 and cutoff point 2 [40].

*Tobacco use problem* as assessed by Fagerstrom Test a score 1 was considered as nicotine dependence [41].

*Adherence to medication* adherence was considered as low, medium and high with a score < 6, 6 and 7, and 8 respectively on 8-Item Morisky 8-item medication adherence scale [42–45].

*Social support* Poor social support, Moderate social support, and Good social support were operationalized at cut-off points 3–8, 9–11, and 12–14 points respectively on the Oslo-3 social support scale [46].

### Data collection procedure

Questionnaires prepared in English and translated to Amharic were used to collect the data. An interviewer based questionnaire was used to collect data regarding common mental disorders using a standardized and valid SRQ questionnaire having 20 items was used to assess common mental disorders [39]. A cutoff point 7 and above was considered for delineating the presence of common mental disorders. SRQ-20 asses common mental symptoms in the past 30 days as Yes/No and Its Amharic version had been validated in Ethiopia [47] and used in several institutions based [48–51] as well community-based studies in Ethiopia [52–54].

Alcohol misuse was assessed using CAGE 4 items questions which had a score of 0–4 and with sensitivity 0.71 and specificity 0.90 at a cutoff point ≥ 2 [55] and used in several previous studies [56–59] for screening people who have problem drinking.

*Nicotine dependence* was assessed by Fagerstrom Test, a score ≥ 1 indicates tobacco use problem reliability coefficient(a) = 0.8 [41]. Morrisk-8 medication adherence scale was used to asses adherence to medication: low-adherence if a score is < 6, medium adherence if a score is 6 and 7, and high adherence if a score is 8 on 8-Item Morisky medication adherence scale [42–45]. Social support as assessed by Oslo-3 social support scale Poor social support, Moderate social support, and Good social support were operationalized at cut-off points 3–8, 9–11, and 12–14 points [46].

### Data quality assurance

The questionnaire was pre-tested on 22 (5%) of the sample at Borumeda hospital 1 week before the actual data collection period. Data was collected by BSc psychiatry nurses after adequate training was given about research aims, procedures, and ethical issues. The collected questionnaire was checked for clarity, consistency, and completeness by the investigators every day and necessary corrections were made before the start of the next day’s work. Double data entry was done for reliability and correctness and computer data cleaning was undertaken.

### Data processing, analysis, and interpretation

Epi-info version 7, was used as a data entry tool and the Statistical Packages for Social Sciences version 20 (SPSS-20) was utilized to analyze data after it was exported. Descriptive statistics (percentages, mean, median, standard deviation and crosstabs) were utilized to summarize common mental disorder and its predictor variables. A logistic regression model was fitted to asses potential risk factors for a common mental disorder. Variables with *p* value < 0.25 in bivariate analysis were pooled into multivariable logistic regression. Odds ratio with 95% CI was employed to measure the strength of association and statistical significance was set at a p-value of < 0.05 in the final model.

## Result

### Socio-demographic characteristics of the respondents

A total of 422 patients with epilepsy on follow-up treatment and evaluations at Felegehiwot Referral Hospital participated in the study with a response rate of 100%. The mean age of the participants was 30.7 years, with a Standard Deviation of 10.28 years and most of, 120 (28.4%) were between the age of 25–34 years. More than half (55.2%) of the respondents were males. A higher proportion (67%) of study participants had urban residency. Nearly two-thirds (62.6%) of the study participants were Muslims. About 229 (54.3%) of the participants were married and nearly one-fourth (25.6%) are farmers (Table 1).

**Table 1:** Sociodemographic characteristics of epileptic patients attending Felegehiwot Referral Hospital epileptic clinic (n = 422), Bahirdar, Ethiopia, February 2019

Characters	Classification	Frequency	Percent	Have no mental distress N (%)	Have mental distress N (%)
Sex	Male	233	55.2	151 (64.8)	82 (35.2)
	Female	189	44.8	120 (63.5)	69 (36.5)
Age	18–24 years	120	28.4	79 (65.8)	41 (34.2)
	25–34 years	170	40.3	115 (67.6)	55 (32.4)
	35–44 years	92	21.8	54 (58.7)	38 (41.3)
	45 and above	40	9.5	17 (42.5)	13 (57.5)
Marital Status	Married	229	54.3	150 (65.5)	79 (34.5)
	Single	176	41.7	111 (63)	65 (37)
	Divorce/separate/widowed	17	4	10 (58.8)	7 (41.2)
Religion	Orthodox	128	30.3	75 (58.6)	53 (41.4)
	Muslim	264	62.6	177 (67)	87 (33)
	Protestant	29	6.9	19 (65.5)	11 (34.5)
Address	Urban	266	63.0	171 (64.3)	95 (35.7)
	Rural	156	37.0	100 (64)	56 (36)
Educational Status	Illiterate	54	12.8	36 (66.7)	18 (33.3)
	Can read and write	71	16.8	42 (59)	29 (41)
	Primary	78	18.5	49 (62.8)	29 (37.2)
	Secondary	146	34.6	103 (70.5)	43 (29.5)
	College and above	73	17.3	41 (56)	32 (44)
Occupational Status	Farmer	108	25.6	69 (63.9)	39 (45.1)
	Gov’ t employers	97	23.0	63 (64.9)	34 (35.1)
	Self-employee	101	23.9	64 (63.4)	37 (36.6)
	Student	93	22.0	60 (64.5)	33 (35.5)
	Other	23	5.5	15 (65.2)	8 (34.8)
Monthly income	< 700	215	50.9	193 (78.8)	22 (20.2)
	700–1499	24	5.7	22 (91.7)	2 (8.3)
	> 1500	183	43.4	163 (89)	20 (11)

### Clinical characteristics of epileptic patients

One hundred ninety-seven (46.7%) of epileptic patients were taking anti-epileptic medication for less than 5 years and the type of anti-epileptic medications most study participants using was phenobarbitone 148 (73.3%). Regarding the controllability of seizure, most 375 (88.8%) had controlled seizure by current medication and did not complain epileptic fits in spite of taking their medications, 40 (9.5%) were having 1–2 seizure attacks while taking medication in the last 30 days and 7 (1.7%) of the respondents had 3–12 seizure attack even though they were on medication (Table 2).

**Table 2:** Clinical characteristics of epileptic patients attending Felegehiwot Referral Hospital epileptic clinic (n = 422), Bahirdar, Ethiopia, February 2019

Variables	Category	Frequency	Percent
Number of seizure attack the last 30 days	1–2	40	9.5
	3–12	7	1.7
	Never	375	88.9
Duration on AED medication	< 5 years	197	46.7
	5–10 years	119	28.2
	> 10 years	106	25.1
Epileptic fits	Yes	42	9.9
	No	380	90.1
History of chronic medical illness	Yes	15	3.6
	No	407	96.4
Lifetime substance use	Yes	186	44.1
	No	236	55.9
Substance use in the last 30 days	Yes	94	22.3
	No	328	77.7
Alcohol misuse	CAGE ≥ 2	36	8.5
	CAGE < 2	386	91.5
Nicotine dependence	Yes (FTND ≥ 1)	30	7.1
	No (FTND < 1)	392	92.9
Medication adherence	Low	394	93.4
	Intermediate	24	5.7
	High adherence	4	0.9
Family history of mental illness	Yes	7	1.7
	No	415	98.3
Social support	Poor	82	19.4
	Moderate	254	60.2
	High	86	20.4

*CAGE* Cut down, Annoyed, Guilty feeling and Eye opener; *FTND* Fagerstrom Test of Nicotine Dependence

### Prevalence of common mental disorders among epileptic patients at Felegehiwot Referral Hospital

**Fig. 1:**
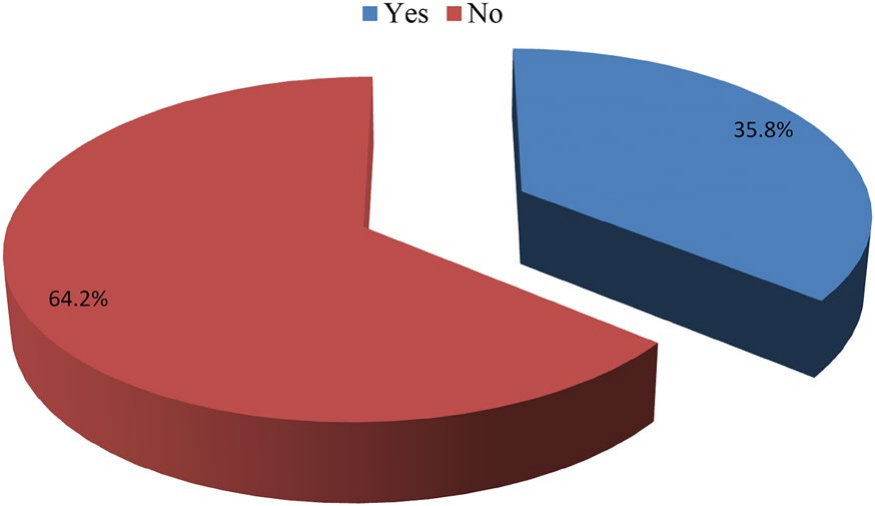
Prevalence of common mental disorders among Epileptic patients attending Felegehiwot Referral Hospital epileptic clinic (n = 422), Bahirdar, Ethiopia, February 2019

A cut-off point 7 on the self-reporting questionnaire-20 was used [39]. epileptic patients who scored 7 and above on the SRQ-20 were categorized as having common mental disorders while those scoring less than 7 on the SRQ-20 classified as not having common mental disorders. The prevalence of the common mental disorder in this study was 35.8% (95% CI 30.8, 40.4) (Fig. 1). The high common mental disorder was observed in the age group 45 years and older (57.5%) and divorced/widowed groups had high CMD (41.2%). The most prominent common mental disorder symptoms in this study were a headache (43.6%), poor appetite (41.5%), poor sleeping conditions (34.6%) and feeling tired all the time (24.6%). Suicidal ideation was complained by 74 (17.5%) of participants (Table 3).

**Table 3:** Common mental disorder symptoms of epileptic patients attending Felegehiwot Referral Hospital epileptic clinic (n = 422), Bahirdar, Ethiopia, February 2019

Characters	Classification	Frequency	Percent
Do you often have headache?	Yes	184	43.6
	No	238	56.4
Is your appetite poor?	Yes	175	41.5
	No	247	58.5
Do you sleep badly?	Yes	146	34.6
	No	276	65.4
Are you easily frightened?	Yes	75	17.8
	No	347	82.2
Do your hands shake?	Yes	60	14.2
	No	362	85.8
Do you feel nervous, tense or worried?	Yes	86	20.4
	No	336	79.6
Is your digestion poor?	Yes	71	16.8
	No	351	83.2
Do you have trouble thinking clearly?	Yes	82	19.4
	No	340	80.6
Do you feel unhappy?	Yes	75	17.8
	No	347	82.2
Do you cry more than usual?	Yes	87	20.6
	No	335	79.4
Do you find it difficult to enjoy your daily activities?	Yes	82	19.4
	No	340	80.6
Do you find difficult to make decision?	Yes	78	18.5
	No	344	81.5
Is your daily work suffering?	Yes	88	20.9
	No	334	79.1
Are you unable play a useful part in life?	Yes	89	21.1
	No	333	78.8
Have lost interest in things?	Yes	78	18.5
	No	344	81.5
Do you feel that you are a worthless person?	Yes	67	84.1
	No	355	15.9
Has the thought of ending your life been on your mind?	Yes	74	17.5
	No	348	82.5
Do you feel tired all the time?	Yes	104	24.6
	No	318	75.4
Are you easily tired?	Yes	80	81
	No	342	19

### Factors associated with common mental disorders among epileptic patients

Variables which pass into multivariate analysis with p-value < 0.2 on bivariate regression were age, marital status, educational level, religion, current epileptic medication, duration of epilepsy, mental illness in family, comorbid medical illness, alcohol dependence, nicotine dependence, medication non adherence and frequency of seizure but only marital status, comorbid medical illness, alcohol dependence, nicotine dependence, medication non adherence were significantly associated in final model with p-value < 0.05.

Participants who were divorced, widowed and separated as a group were about two times more likely (AOR = 1.95, 95% CI 1.67, 5.67) to develop the common mental disorder as compared to participants with a sustained marital relationship. The odds of study participants to develop a common mental disorder in the presence of comorbid medical illness was three times higher relative to participants with no comorbid medical condition (AOR = 2.99, 95% CI 1.95, 9.39).

Alcohol dependence was significantly associated with a common mental disorder. Participants with Alcohol dependence as measured by CAGE ≥ 2 were 2.2 times more likely to have mental disorder as compared to those who score CAGE < 2(AOR = 2.20, 95% CI 1.78, 3.93). The odds of developing mental disorder among nicotine-dependent participants was slightly higher (AOR = 1.52, 95% CI 1.46, 4.62) relative to those participants with no nicotine dependence. Poor medication adherence was 2 times higher a risk factor to predispose to common mental disorder than good medication adherence (AOR = 1.9, 95% CI 1.72, 3.56) (Table 4).

**Table 4:** Bivariate and multivariable Logistic Regression analysis Result of the factors for common mental disorder among epileptic patients at Felegehiwot Referral Hospital, Bahirdar, Ethiopia, 2019 (n = 422)

Explanatory variable	CMD		COR (95% CI)	AOR (95% CI)
	Yes	No		
Marital status				
Married	79	150	1.00	1.00
Single	65	111	1.12 (0.74, 1.67)	1.37 (0.76, 2.44)
Divorced/separated	7	10	1.34 (1.17, 3.63)	1.95 (1.67, 5.67)^a^
Age of participants				
18–24	41	79	1.00	1.00
25–34	55	115	0.9 (0.56,1.52)	1.04 (0.54, 2.03)
35–44	38	54	1.36 (0.78, 2.38)	1.37 (0.57,3.18)
45 and above	13	17	1.43 (0.68, 2.96)	1.8 (0.67, 4.85)
Duration epileptic rt.				
< 5 Years	79	118	1.00	1.00
5–10 Years	41	78	1.62 (0.96, 2.69)	0.78 (0.46, 1.33)
> 10 years	31	41	1.27 (0.73, 2.24)	0.50 (0.27, 0.91)
Type of AEM				
Phenobarbitone	110	190	1.00	1.00
Phenytoine	30	48	1.74 (0.56,5.52)	1.18 (0.63, 2.19)
Carbamazipine	7	21	1.88 (0.55,6.35)	0.55 (0.21, 1.42)
Valporic acid	4	12	1.02 (0.24, 4.13)	0.73 (0.22,2.46)
Comorbid illness				
Yes	8	7	2.1 (1.75, 5.93)	2.99 (1.95, 9.39)^b^
No	143	264	1.00	1.00
Alcohol misuse				
CAGE score ≥ 2	20	131	2.43 (1.91, 4.85)	2.20 (1.78, 3.93)^b^
CAGE score < 2	16	255	1.00	1.00
Nicotine dependence				
FTND score ≥ 1	14	16	1.63 (1.77, 6.44)	1.52 (1.46, 4.62)^b^
FTND score < 1	137	255	1.00	1.00
Medication adherence				
Good	11	29	1.00	1.00
Poor	140	242	1.53 (1.24, 3.15)	1.9 (1.72, 3.56)^b^
Mental illness in family				
Yes	5	2	4.6 (0.88, 24.00)	4.88 (0.79,29.8)
No	146	269	1.00	1.00

^a^p-value < 0.05, ^b^p-value < 0.01, Model Chi square = 2.96, df = 8 and sig = 0.937

## Discussion

Common mental disorders were prevalent in 35.8% of participants in this study. Comorbidity of a medical condition, nicotine dependence, alcohol dependence, medication non-adherence, and divorce/widow were the factors significantly associated with common mental disorders. This magnitude implied that large numbers of epileptic patients had not yet received an adequate intervention for psychiatric disorders specifically for a common mental disorder. So this result revealed that intervention has to be given for such comorbid-psychiatric problems in the epileptic clinic. Moreover, this finding strengthens the need for linkage of services between a psychiatric and neurological service for epileptic patients.

The prevalence of Common mental disorder in this study was in line with studies conducted so far in the USA (36.5%) [26], two Europe studies (37%) [60], (28.6%) [61], Iceland (35.5%) [62] and Ethiopia (35%) [63].

On the other hand, finding of current study was lower than the studies in Brazil (54.1%) [64], USA (70%) [65], (47%) [66] and (78%) [67], Netherlands (75.2%) [20], Iceland (54.8%) [22], Europe (54.8%) [68] and Italy (80%) [69], Ethiopia (45%) [28].The variation in prevalence might be due to the difference in sample size and assessment instruments used. Only 94 participants were included in Brazilian study [64], 60 participants in USA [65], 131 participants USA [67], 88 participants in Europe [68], and 38 participants in Italy [69] and 209 participants in the Netherlands study [20]. Methodological differences like DSM-IV in Brazilian [64], Iceland [22] and USA studies [65], Kessler-6 in the USA [66] and health style interview in the USA [67], CIDI in Netherlands study [20] would also contribute to the difference. Moreover, the study population in which the current study asses all forms of epilepsy but only temporal lobe epilepsy in other studies that had a higher risk of mental disorders [70–72] might cause the variation.

The result of the study was higher than the studies conducted in Canada (23.5%) [14], in northern Sweden (5.9%) [73], in two European studies (11.2%) and (29%) [74, 75]. The different would be attributed to variation in sample size. In the northern Sweden study, a larger sample size of 713 patients with epilepsy was included. The difference in assessment instruments; Hospital anxiety and depression scale was used in European study [74], CIDI was used in Canadian [14] study and unstructured psychiatric interview in European study [73] might also contribute to the variation in the prevalence of the common mental disorder.

Divorce/separation was associated with CMD in this study, which was two times higher risk to develop common mental disorders as compared to married study participants who live together. This was supported by the study done at Emanuel specialized hospital, Addis Ababa, Ethiopia [63] and other studies outside Ethiopia [32, 76]. Divorce is a major psychosocial stressor predisposing to chronic stress which might disturb the social and emotional domains finally leading to common mental disorders.

Comorbidity of medical condition was also associated with a common mental disorder, three times higher risk as compared to participants with no comorbid medical illness. The finding is consistent in previous studies [25, 32] This might be due to medical conditions affecting the quality of life epilepsy patients as supported with a study [77].

Being alcohol dependence had a 2.2 times higher risk of developing a common mental disorder as compared with epileptic patients who had no alcohol dependence. This was supported by comparable studies in the USA [78, 79], New Zealand [80]. The reason might be alcohol consumption causing increased GABAergic neurotransmission and nervous system depression since GABA is inhibitory neurotransmitter [81]. Alcohol might also reduce seizure threshold and increase seizure frequency by affecting calcium and chloride flux through the ion‐gated glutamate and GABA receptors [82] and this might affect patients’ quality of life.

Having poor medication adherence was two times a higher risk factor for common mental disorder than good medication adherence. This was supported by a study outside Ethiopia [83] and in Ethiopia [28]. Non-adherence will lead to reduced seizure control, lowered quality of life, decreased productivity, seizure-related job loss [84] and recurrence of seizures might complicate to mental disorders [85] and since bidirectional interaction between epilepsy and mental disorders; up to 60% of epileptic patients develop depression and depression increase the risk of epilepsy [86, 87].

Nicotine dependence was a risk factor for CMD in this study, which was 1.5 times higher risk to develop common mental disorders as compared to participants who have no nicotine dependence. This was supported by a study finding that asses smoking as a risk factor for major depression [88] and mental illness in general [89] The reason might be due to cigarette smoking increases risk of seizure recurrence [90] which might, in turn, lowers quality of life of epileptic patients.

## Conclusion

This study found that common mental distress among epileptic patients was high. Divorce from socio-demographic variables, clinical variables such as comorbidity of medical illness, medication non-adherence and substance-related factors like alcohol misuse and nicotine dependence were risk factors for common mental disorder in this study. Early screening and treatment of epileptic patients have to be a routine activity to be conducted in epileptic clinics.

## Data Availability

The datasets used and/or analyzed during the current study are available from the corresponding authors on reasonable request.

## Abbreviations

AED: anti-epileptic drugs
CBE: community based education
CIDI: Composite International Diagnostic Interview
CMD: common mental disorders
EEG: electro encephalon gram
GABA: γ‐amino butyric acid
OPD: out patient department
PWE: patient with epilepsy
SRQ: self reporting questionnaires
USA: United States of America
WHO: World Health Organization
